# Chemical Constituents of Root Barks of *Gnidia involucrata* and Evaluation for Antibacterial and Antioxidant Activities

**DOI:** 10.1155/2019/8486214

**Published:** 2019-08-14

**Authors:** Abera Kalbessa, Aman Dekebo, Hailemichael Tesso, Teshome Abdo, Negera Abdissa, Yadessa Melaku

**Affiliations:** ^1^Chemistry Department, School of Applied Natural Sciences, Adama Science and Technology University, Adama, Ethiopia; ^2^Chemistry Department, College of Natural Sciences, Jimma University, Jimma, Ethiopia

## Abstract

The genus *Gnidia*, with species close to 152, is traditionally used to treat wide ranges of ailments in humans and animals. *Gnidia involucrata* is one of the species found in Ethiopia and traditionally used as a laxative, antirheumatic agent, insecticide, antibacterial agent, and antimalarial agent. In view of its traditional use, the root bark was sequentially extracted with *n*-hexane, EtOAc, and MeOH to afford 0.78%, 4%, and 6% crude extracts, respectively. The chromatographic separation of the EtOAc extract using silica gel column chromatography yielded three pure compounds: tetratriacontanyl caffeate (**1**), 12-*O*-dodeca-2,4-dienoylphorbol-13-acetate (**2**), and naringenin (**3**). This is the first report of the isolation of **1** and its kind from the genus and most probably from the Thymelaeaceae family. The structures of these compounds were characterized and identified by NMR and mass spectrometric analyses and comparison with literature data. The EtOAc extract and isolated compounds were assessed for their *in vitro* antibacterial and antioxidant activities. The EtOAc extract (1.5 mg/mL) showed significant inhibitory activity against *S. aureus*, *E. coli*, *P. mirabilis*, and *K. pneumonia* bacterial strains with the highest inhibition zone observed against *S. aureus* (23 mm), which is even greater than that of the reference drug ciprofloxacin (22 mm). However, the inhibition displayed on these bacterial strains for the three pure compounds was marginal with variable degrees of potency between the compounds. The better activity of the crude extract could be due to the synergistic interactions of several phytochemicals present in the extract, which cannot be the case when pure compounds are evaluated alone. The antioxidant activities of the extracts and isolated compounds were evaluated using DPPH and ferric thiocyanate methods. The EtOAc and MeOH extracts and compounds **1** and **2** were found to inhibit the DPPH radical by 70.7, 66.9, 85.8, and 52.8%, respectively. The EtOAc extract and compound **1** inhibited peroxidation of lipids by 84 and 86%, respectively. The radical scavenging displayed by compound **1** was significant compared with that displayed by ascorbic acid, indicating the strong antilipid peroxidation potential of the extract of root barks of *G. involucrata*. Therefore, the extracts of the root bark of *G. involucrata* can be used as a remedy in combating diseases caused by bacteria and free radicals provided that further comprehensive evaluation could be recommended for the conclusive decision on potential candidacy of this plant.

## 1. Introduction

Infectious diseases caused by bacteria remain a serious public health problem in developing countries [1, 2]. The problem is exacerbated by the development of bacterial resistance to currently used antibacterial drugs [3, 4]. Among the major strategies to alleviate the current situation is to search for new antibacterial agents from natural sources as an alternative to synthetic antibiotics. Plants are known to produce a variety of secondary metabolites to protect themselves against a variety of their own pathogens, and therefore, those with documented traditional use appear to be a rich source of new antibacterial and antioxidant drugs. This prompts a revival in search of antibacterial and antioxidant agents from natural products.

*Gnidia* (of family Thymelaeaceae) is a widespread genus comprising about 152 species [5]. Although *Gnidia* species are being recognized for their ornamental value due to their bright and colorful flowers [6], they are also known for their use in traditional medicinal practice [7–9] and for their economic value as the flowers of the species are employed for dying leather [10]. *Gnidia involucrata* is one of the species growing in Ethiopia and locally called *shuntura* in Afan Oromo and *Yezngero telba* in Amharic. It is a perennial grass or subshrub that is being used in Ethiopia for the treatment of a wide array of diseases including malaria, rheumatism, and stomach parasite [11]. Its use as an insecticide and an insect repellant and also for the treatment of intestinal pain, mental problems, sexually transmitted diseases, and tuberculosis has been documented [12, 13]. In Ethiopia, the roots are used as a laxative and vermifuge [8]. In folk medicine, the roots are used to reduce the size of the vaginal orifice and as fish poison. Previous pharmacological reports indicate a variety of therapeutic uses such as antifungal agents [14], *α*-amylase inhibitors [15], and anti-inflammatory and gastroprotective agents [16].

Some species in the genus *Gnidia* were reported to contain secondary metabolites including manniflavanone, kaempferol-3-*O*-glucoside, *Gnidia* biflavonoid, vitexin, isovitexin, isoorientin, mangiferin, 2,3,4′,5,6-pentahydroxybenzophenone-4-*C*-glucoside, 2,4′,6-trihydroxy-4-methoxybenzophenone-2-*O*-glucoside, mhakoside A, and yuankanin [17, 18]. Despite the enormous traditional use of this species against various diseases, there is no information on the chemistry and antibacterial and antioxidant studies of the root barks of *G. involucrata*. Hence, in this study, we report for the first time the isolation of three compounds from the barks of *G. involucrata.* The antibacterial and antioxidant activities of the root bark extracts and isolated compounds are also reported herein for the first time.

## 2. Materials and Methods

### 2.1. Plant Material

The root barks of *G. involucrata* were collected in December 2016 from Kunde Gerasu Kebele, Meta Wolkite Woreda, West Shewa, Oromia, Ethiopia. Identification and authentication of the plant specimen was done at the National Herbarium of Addis Ababa University by Prof. Legesse Negash, and the voucher specimen (AB-002/2016) has been deposited.

### 2.2. Extraction and Isolation

The air-dried root barks (300 g) of *G. involucrata* were milled into powder and then sequentially extracted with 1.5 L of *n*-hexane, EtOAc, and methanol each for 72 hours with occasional shaking at room temperature. The extracts were filtered and concentrated under reduced pressure using a rotary evaporator at 40°C to yield 2.4 g (0.8%), 12 g (4%), and 18 g (6%), respectively. The ethyl acetate extract (10 g) was adsorbed and fractionated over silica gel (200 g) column chromatography with increasing polarity of EtOAc in *n*-hexane to afford *ca*. 200 mL of each of 22 major fractions. The first four fractions were collected with 100% *n*-hexane. Fr5-6, Fr7, Fr8-9, Fr10-11, Fr12, Fr15-16, Fr17-18, and Fr19-20 were eluted with 9 : 1, 4 : 1, 7 : 3, 3 : 2, 1 : 1, 2 : 3, 1 : 4, and 1 : 9 *n*-hexane : EtOAc, respectively. The last fractions, Fr21-22, were collected with 100% EtOAc. Fractions 12 (875 mg) after silica gel column chromatography with 4 : 1 *n*-hexane : EtOAc as an eluent furnished compound **1** (46 mg). Fractions 17-18 (433 mg) showed three spots on TLC, which were combined and subjected to column chromatography on silica gel with 85 : 15 CH_2_Cl_2_ : MeOH as an eluent to give compound **2** (66 mg) and compound **3** (23 mg).

### 2.3. Antioxidant Activities

#### 2.3.1. DPPH Radical-Scavenging Assay

The radical-scavenging activities of the extracts and isolated compounds were determined using the DPPH method [19, 20]. Four different concentrations of the methanol extract were mixed with 0.04% DPPH in MeOH to furnish 100, 50, 25, and 12 *μ*g/mL. The resulting solutions were placed in an oven at 37°C for 30 minutes and subjected to a UV-Vis spectrophotometer to record absorbance at 517 nm. Likewise, the radical-scavenging activities of the EtOAc extract and isolated compounds were evaluated following the same protocol. Ascorbic acid was used as a positive control. The percentage inhibition was calculated using the following formula:

(1) $$\begin{array}{c} \%\text{ inhibition }=\frac{A_{\text{control}}-A_{\text{extract}}}{A_{\text{control}}}\times 100, \end{array}$$

where *A*_control_ is the absorbance of the DPPH solution and *A*_extract_ is the absorbance of the test sample (DPPH solution plus sample).

#### 2.3.2. Ferric Thiocyanate Method

The antilipid peroxidation potential of the extracts and isolated compounds was evaluated according to the method of Nagatsu [21]. 0.1 mg of each of the extracts and pure compounds of *G. involucrata*, 100 *μ*L of linoleic acid, EtOH (5 mL), and phosphate buffer (5 mL, 0.05 M, pH = 7) in water were separately added into a vial and incubated at 40°C in an oven. After 24 h, 0.1 mL from each was taken and added into a vial containing 75% of aqueous EtOH (7 mL), 30% of NH_4_SCN (0.15 mL), and 0.15 mL of 0.02 M FeCl_2_ in 3.5% HCl. Each was then subjected to UV-Vis spectrophotometry to record the absorbance at 500 nm. Absorbance of the blank and ascorbic acid was recorded in the same fashion. The percentage inhibition using the ferric thiocyante method is calculated according to the following formula:

(2) $$\begin{array}{c} \text{Percentage inhibition}=100-\left(\frac{A_{\text{s}}}{A_{\text{b}}}\times 100\right)\%, \end{array}$$

where *A*_s_ is absorbance of the sample and *A*_b_ is absorbance of the blank [22].

### 2.4. Antibacterial Activities

The antibacterial activities of the ethyl acetate extract and isolated compounds from the barks of *G. involucrata* were tested against four bacterial strains using the disc diffusion method [23]. The American Type Culture Collection (ATCC) bacterial strains were obtained from Oromia Public Health Research, Capacity Building and Quality Assurance Laboratory Center, Adama, Ethiopia. The antibacterial activities of all samples were tested against three Gram-negative (*Escherichia coli* (ATCC 25922), *Proteus mirabilis* (ATCC 35659), and *Klebsiella pneumonia* (ATCC 700603)) and one Gram-positive (*Staphylococcus aureus* (ATCC 25923)) bacterial strains using the Mueller–Hinton agar (MHA) medium.

The test bacterial species were transferred from the stock cultures, and microbial suspensions were prepared in a nutrient broth for 24 h at 37°C until the turbidity of bacterial suspensions reached 1.5 × 10^8^ CFU·mL^−1^ by comparison with the 0.5 McFarland Standard. The disc diffusion assay was carried out by swabbing each test strain on the Mueller–Hinton (MH) agar plate using the 1/10 dilution of the microbial suspensions. Sterile paper discs (Whatman No.1 filter paper) impregnated with sterile 20 *μ*L (1.5 mg/mL) concentrations of the sample were placed onto the surface of the agar plate with equal distance from each other. A disc impregnated with sterile solvent (water) was taken as a negative control. Ciprofloxacin was used as the standard drug (positive control). After overnight incubation at 37°C, zones of inhibition around the disc were observed. Antimicrobial activity (*x*) was then characterized and classified based on the inhibition growth zone diameters and described as slight (*x* < 4 mm diameter), medium (*x* = 4–8 mm), high (*x* = 8–12 mm), and very high (*x* > 12 mm) [24].

## 3. Results and Discussion

### 3.1. Characterization of Isolated Compounds

**Figure 1:**
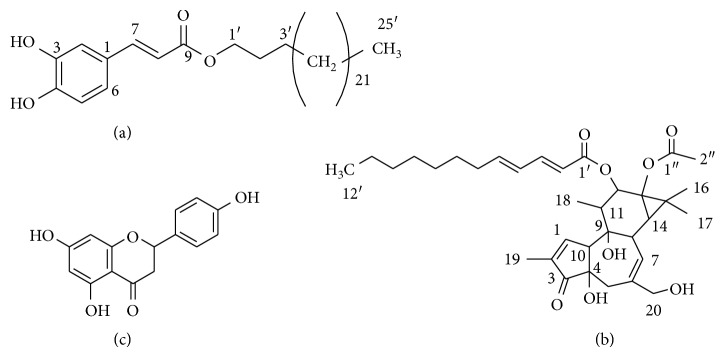
Structures of isolated compounds **1** (a), **2** (b), and **3** (c).

The root bark of *G. involucrata* was sequentially extracted with *n*-hexane, EtOAc, and MeOH. The EtOAc extract was subjected to column chromatography for further purification following its better radical-scavenging activity and peroxide formation inhibition and afforded three compounds **1**–**3** (Figure 1). The characterizations of the spectroscopic data for the identification of the structure of these compounds are detailed below.

Compound **1** was isolated as a white crystal from the EtOAc extract. Its TLC showed a spot at *R*_f_ 0.42 with *n*-hexane : EtOAc (7 : 3) as a mobile phase which was visualized after dipping in vanillin/H_2_SO_4_. The ESI-MS provided a molecular ion peak at *m/z* 553 for [M + Na]^+^ and *m/z* 1083 for [2 M + Na]^+^, both corresponding to the molecular formula of C_34_H_58_O_4_, indicating six degrees of unsaturation. In the IR spectrum, the sharp absorption at 1684 cm^−1^ was attributed to the presence of the conjugated carbonyl group and the broad band at 3487 and 3328 cm^−1^ to the hydroxyl groups.

The ^1^H-NMR spectrum (Table 1) indicated the presence of three protons in the aromatic region at *δ*_H_ 6.88 (1H, d, *J* = 8.0 Hz), 7.04 (1H, dd, *J* = 1.8 and 8.0 Hz), and 7.13 (1H, pseudosinglet), which were assigned to H-5, H-6, and H-2, respectively, based on their coupling constants and mutual couplings. It also exhibited signals corresponding to a pair of doublets at *δ*_H_ 6.25 and 7.57 with the vicinal coupling constant ^3^ *J*_HH_ = 15.6 Hz. This indicated a *trans* geometry, and the signals were assigned to H-8 and H-7, respectively. These spectral features observed in the aromatic region in combination with the *trans* double-bond protons, which are *peri* to the carbonyl group (from the chemical shift value), were a clear indication of the presence of the caffeic acid moiety. The signal at *δ*_H_ 4.19 (2H, t) was apparent for the existence of protons on oxygenated methylene carbon. The spectrum also showed signals accounting for 34 hydrogen atoms centered at *δ*_H_ 1.27, indicating the presence of protons on many methylene groups. The signal at *δ*_H_ 1.73 (4H) and *δ*_H_ 0.90 (10H) revealed the presence of hydrogen atoms on two and five methylene groups, respectively. The presence of terminal methyl protons was evident at *δ*_H_ 0.88.

The proton-decoupled ^13^C-NMR spectrum (Table 1) of compound **1** with the aid of DEPT-135 showed signals corresponding to 34 carbon atoms including five methine groups (two olefinic and three aromatic), twenty-four methylene groups (one oxygenated), four quaternary carbon atoms (one carbonyl carbon), and a methyl carbon atom. The highly downfield-shifted carbon signal at *δ*_C_ 168.0 and the two olefinic carbon atoms at *δ*_C_ 143.9 and 115.5 were evident for the presence of the *α*,*β*-unsaturated carbonyl group of the caffeic acid moiety. The signal at *δ*_C_ 64.9 for the oxygenated methylene carbon and long aliphatic chain revealed the presence of caffeic acid ester. Based on these spectroscopic data and the literature information, the structure of compound **1** was concluded to be pentacontanyl caffeate, previously reported from the leaves of *Artemisia argyi* except for the long chain alcohol component [25].

The spectral data of compound **1** are in agreement with the structure shown in Figure 1.

Compound **2** was obtained as a yellow crystal with an *R*_f_ value of 0.56 (in 15% methanol in dichloromethane as a mobile phase), which was visualized after dipping in iodine solution. Its molecular formula C_34_H_48_O_8_ was deduced from ESI-MS, showing a peak at *m*/*z* 1191 for [2 M + Na]^+^, indicating eleven indices of hydrogen deficiency. The IR spectrum showed a stretching band due to the presence of hydroxyl at 3415 cm^−1^. The strong absorption bands at 2925 cm^−1^ and 1714 cm^−1^ were evident for the presence of the C-H stretching of alkyl groups and *α*,*β*-conjugated carbonyl, respectively, whereas the bands at 1641 and 1259 cm^−1^ were apparent for C=C and C-O stretching, respectively.

The ^1^H-NMR spectrum (Table 2) showed signals for four olefinic protons at *δ*_H_ 5.76 (1H, d, *J* = 15.2 Hz, H-2′), 6.16 (1H, m, H-5′), 5.69 (1H, br.s, H-4′), and 7.21 (1H, dd, *J* = 10 Hz, H-3′). The downfield-shifted olefinic proton signal at *δ*_H_ 7.58 was assigned to proton at C-1, *beta* to the carbonyl group. Signals in the ^1^H-NMR spectrum at *δ*_H_ 4.02 and 3.95 were due to the presence of oxygenated methylene protons. The signals at *δ*_H_ 2.47 and 2.59 also accounted for the presence of methylene protons in different environments. The spectrum showed the presence of six methyl protons at *δ*_H_ 2.10 (3H, s H-2″), 1.74 (3H, s, H-19), 1.25 (3H, s, H-16), 1.2 (3H, s, H-17), and 0.88 (6H, t, H-12′ and H-18). The spectrum also showed signals accounting for 8 hydrogen atoms centered at *δ*_H_ 1.27, indicating the presence of protons on many overlapping methylene groups.

The proton-decoupled ^13^C-NMR spectrum with the aid of DEPT-135 showed the presence of eleven methine groups, eight methylene groups, 6 six methyl groups, and nine quaternary carbons. The downfield-shifted signal observed at *δ*_C_ 209.3 was evident for the presence of the *α*,*β*-unsaturated carbonyl group. In fact, the high chemical shift value for this conjugated carbonyl (*δ*_C_ 209.3) could be due to the deshielding and steric effects of the *di-alpha* substituent, which could not allow the planarity of the carbonyl group with the double bond. The signals at *δ* 173.9 and 167.2 were apparent for the presence of two ester moieties. The signals observed at *δ*_C_ 118.8, 145.3, 128.3, and 145.7 were evident for the olefinic groups. The other signals due to quaternary carbons were observed at *δ*_C_ 132.8, 73.7, 140.7, 78.4, 65.7, and 25.7. The ^13^C-NMR spectral data of compound **2** were very similar to those reported for prostratin Q, which was isolated from *Wikstroemia chamaedaphne* [26], except for additional methylene groups to the long chain aliphatic ester (Table 2), and aquimavitalin extracted from *Aquilaria malaccensis*, except for the length of the fatty acid moiety and number of olefinic protons [27]. Therefore, based on this spectroscopic evidence, compound **2** was identified as prostratin (12-*O*-phorbol-13-acetate).

Prostratin (12-*O*-phorbol-13-acetate), a nontumor-promoting phorbol ester, reported to inhibit HIV-1 cell entry and replication, blocks completion of reverse transcription of the HIV-1 genome in lymphoid tissue and infection of CD4^+^ T lymphocytes and at the same time reactivates virus from latency, restricts primary resting of CD4^+^ T-cell susceptibility to HIV-1 infection in primary blood mononuclear cells (PBMCs) and in lymphoid tissue [28, 29], has antireplicative and anticytopathic activities against HIV [30], and inhibits ornithine decarboxylase induction, *edema*, and hyperplasia [31]. 12-*O*-Dodeca-2,4-dienoylphorbol-13-acetate isolated in this work from this plant may exhibit activities displayed by those compounds containing the prostratin nucleus. Therefore, the presence of this compound adds one positive attribute to the root barks of this plant.

Compound **3** was obtained as pale yellow needles. Its TLC profile showed a spot at *R*_f_ value 0.60 in 15% CH_2_Cl_2_ in MeOH as a mobile phase which was visualized after dipping in iodine. The IR spectrum of compound **3** showed the presence of hydroxyl and carbonyl stretching at 3328 and 1641 cm^−1^, respectively. The ^13^C-NMR spectral data with the aid of DEPT-135 indicated a total of fifteen carbon atoms including seven methine groups, one methylene group, and seven quaternary carbon atoms. The downfield signal observed at *δ*_C_ 197.2 was evident for the presence of the carbonyl group. The signal at *δ*_C_ 47.9 was indicative of the presence of aliphatic carbon in the ring. Other signals were observed at *δ*_C_ 83.4, 164.5, 96.1, 166.1, 94.8, 163.3, 101.9, 128.5, 129, 114.9, and 157.6. Of these, the signals situated at *δ*_C_ 197.2, 164.5, 166.1, 163.3, 101.9, 128.5, and 157.6 accounted for quaternary carbons. These spectral data are consistent with the spectral data reported for naringenin, previously reported from *Nyctanthes arbortristis* [32].

### 3.2. Antioxidant Activities

#### 3.2.1. DPPH Radical-Scavenging Assay

The extracts and isolated compounds were assessed for their radical-scavenging activities using DPPH. The results showed that the ethyl acetate and methanol extracts and compound **1** and compound **2** inhibited the DPPH radical by 70.7, 66.9, 85.8, and 52.8% at 100 *μ*g/mL, respectively (Table 3). The IC_50_ parameter is also applied to express the antioxidant activity of compounds or mixtures by the DPPH [33]. In view of this, the radical-scavenging activity of the samples analyzed in this work was also expressed in terms of IC_50_ value. The results showed that the EtOAc extract, the methanol extract, and compounds **1** and **2** displayed IC_50_ values of 7.9, 17.7, 73.0, and 84.9, respectively. The best activity was shown by compound **1** which likely accounts for the activity displayed by the EtOAc extract. The radical-scavenging activity of compound **1** was found comparable with that of ascorbic acid which inhibits the radical by 90%. The activity displayed by compound **1** is likely due to the presence of phenolic hydroxyl groups. Therefore, ethyl acetate extract and compound **1** may be used for the treatment of various life-threatening diseases caused by free radicals.

#### 3.2.2. Ferric Thiocyanate Methods

The degree of lipid peroxidation which was evaluated using the ferric thiocyanate method can be used to measure the antioxidant potential of compounds or extracts. Table 3 shows the results of the antilipid peroxide formation of the root bark extracts and constituents of *G. involucrata*.

As depicted in Table 3, the EtOAc extract and compound **1** inhibit peroxide formation by 84 and 86%, respectively, demonstrating their potential in preventing the formation of lipid peroxides. The results turned out to be comparable with those of ascorbic acid which inhibited the DPPH radical by 87%. On the contrary, the MeOH extract and compound **2** were shown to have low ability of inhibiting peroxide formation compared with the natural antioxidant. This indicates that the antioxidant compound of the root bark extracts of *G. involucrata* resides in the EtOAc extract, with the main active ingredient found to be compound **1**.

### 3.3. Antibacterial Activities

The antibacterial activity of the EtOAc extract and isolated compounds **1**–**3** of the root barks of *G. involucrata* was investigated using the agar well diffusion method, against some selected human pathogens, with the results being presented in Table 4.

The extract displayed a broad range of antibacterial activities against all tested pathogens used in this study including *S*. *aureus*, *E*. *coli*, *P*. *mirabilis*, and *Klebsiella pneumonia* with inhibition zones of 23, 14, 12, and 12 mm, respectively. The activity was pronounceable against *S*. *aureus* compared to ciprofloxacin used as the standard antibiotic. The wide zone of inhibition of the EtOAc extract showed that it had great potential as a remedy for infectious diseases caused by bacterial pathogens. Among the isolated compounds, compound **2** was found active against *S*. *aureus* and *E*. *coli* with inhibition zones of about 8 and 11 mm, respectively. On the contrary, compound **3** displayed modest activities against *P*. *mirabilis*, *E*. *coli*, and *K*. *pneumonia* with inhibition zones of 7, 12, and 8 mm, respectively. The better activity of the crude extract could be due to the synergistic interactions of several phytochemicals present in the extract, which cannot be the case when pure compounds are evaluated alone.

## 4. Conclusion

The ethyl acetate extract after silica gel column chromatography furnished three compounds identified as pentacontanyl caffeate (**1**), 12-*O*-dodeca-2,4-dienoylphorbol-13-acetate (**2**), and naringenin (**3**). This is the first report of caffeic acid ester derivative (**1**) and its kind from the genus *Gnidia*. The work presented herein has also demonstrated that the EtOAc extract of the root bark of *G. involucrata* had strong antibacterial activity compared to ciprofloxacin used as a positive control. The antioxidant activities displayed by the EtOAc extract and compound **1** were significant compared with those displayed by ascorbic acid, indicating the potential of the root barks of this species as natural antioxidants. Therefore, the biological activities displayed by the EtOAc extract and isolated compounds from the root bark of *G. involucrata* corroborate the traditional use of this plant against various ailments caused by bacteria. Further comprehensive evaluations including *in vivo* antibacterial activity tests are recommended for conclusive decision on potential candidacy of the plant for formulation and medicinal uses.

## Figures and Tables

**Table 1:** ^1^H- and ^13^C-NMR (CDCl_3_) spectral data of compound **1** and data of closely related compounds in the literature [20].

No.	^1^H-NMR data of compound **1**	^1^H-NMR spectral data reported in the literature [25]	^13^C-NMR data of compound **1**	^13^C-NMR spectral data reported in the literature [25]
1	—		127.4	127.6
2	7.13 (1H, br.s)	7.16 (1H, d, *J* = 1.8)	114.4	115.2
3	—	—	145.0	146.2
4	—	—	146.5	148.6
5	6.88 (1H, d, *J* = 8.0 Hz)	6.87 (1H, d, *J* = 8.0)	115.4	116.3
6	7.04 (1H, dd, *J* = 1.8 and 8.0 Hz)	7.04 (1H, dd, *J* = 8.0 and 1.8)	122.3	122.4
7	7.57 (1H, d, *J* = 15.6)	7.53 (1H, d, *J* = 15.9)	143.9	145.5
8	6.25 (1H, d, *J* = 15.6)	6.28 (1H, d, *J* = 15.9)	115.5	115.8
9	—		168.0	167.4
1′	4.19 (2H, t)	4.14 (2H, t, *J* = 6.7)	64.9	64.6
2′	1.73 (2H, m)	1.68 (m)	31.9	32.6
3′	1.73 (2H, m)		29.7	NR
4′–24′	1.27–0.88 (42H)		29.7–22.6	NR
25′	0.88 (3H, t)		14.1	14.3

NR: not reported.

**Table 2:** ^1^H- and ^13^C-NMR (CDCl_3_) spectral data of compound **2** and literature reported for closely related compounds [26].

No.	^1^H-NMR spectral data of compound **2**	^1^H-NMR literature data [26]	^13^C-NMR spectral data of compound **2**	^13^C-NMR literature data [26]
1	7.58 (1H, s)	7.57 (1H, s)	160.8	161
2	—	—	132.8	133
3	—	—	209.3	209.1
4	—	—	73.7	74
5(a)	2.47 (1H, d)	2.46 (1H, d)	38.4	38.9
5(b)	2.59 (1H, d)	2.53 (1H, d)		
6	—		140.7	140.7
7	5.69 (1H, br.s)	5.66 (1H, d)	129.2	129.5
8	3.29 (1H, m)	3.22 (1H, dd)	38.9	39.3
9	—		78.4	78.4
10	3.24 (1H, br.s)	3.23 (1H, s)	56.0	56.4
11	2.16 (1H, m)	2.15 (1H, m)	43.0	43.4
12	5.43 (1H, d)	5.44 (1H, d)	76.6	76.8
13	—		65.7	66
14	1.09 (1H, d)	1.08 (1H, d)	36.3	36.6
15	—		25.7	26
16	1.25 (3H, s)	1.24 (3H, s)	23.8	24
17	1.2 (3H, s)	1.19 (3H, s)	16.8	17
18	0.88 (3H, d)	0.87 (3H, d)	14.4	14.6
19	1.74 (3H, s)	1.75 (3H, d)	10.1	10.3
20(a)	3.95 (1H, d)	3.97 (1H, d)	68.0	68.3
20(b)	4.02 (1H, d)	4.02 (1H, d)		
1′	—		167.2	167.3
2′	5.76 (1H, d)	5.76 (1H, d)	118.8	119.1
3′	7.21 (1H, dd)	7.21 (1H, dd)	145.7	145.8
4′	5.69 (1H, br.s)	6.16 (1H, dd)	128.3	128.5
5′	6.16 (1H, m)	6.13 (1H, m)	145.3	145.5
6′	2.14 (2H, m)	2.13 (2H, m)	33.0	33.3
7′	1.40 (2H, m)	1.41 (2H, m)	32.9	28.6
8′–11′	1.27 (8H, m)	1.28 (4H, m)	31.8–22.6	31.6
12′	0.88 (3H, t)	0.86 (3H, t)	14.1	14.2
1″			173.9	174
2″	2.10 (3H, s)	2.08 (3H, s)	21.1	21.3

**Table 3:** DPPH radical-scavenging and antilipid peroxidation activities of extracts (EtOAc and MeOH) and isolated compounds (**1** and **2**).

Samples	% DPPH inhibition				Antilipid peroxidation inhibition (%)
	100 *μ*g/mL	50 *μ*g/mL	25 *μ*g/mL	12.5 *μ*g/mL	
EtOAc extract	70.7 ± 0.02	64.1 ± 0.04	57.5 ± 0.03	46.2 ± 0.05	84 ± 0.04
MeOH extract	66.9 ± 0.01	61.3 ± 0.02	54.7 ± 0.01	41.5 ± 0.10	45 ± 0.06
Compound **1**	85.8 ± 0.03	79.2 ± 0.05	73.4 ± 0.01	65.0 ± 0.02	86 ± 0.01
Compound **2**	52.8 ± 0.01	41.5 ± 0.01	34.9 ± 0.02	16.9 ± 0.03	33 ± 0.09
Ascorbic acid	90 ± 0.02				87 ± 0.02

Data are reported as mean ± SEM; ascorbic acid was used as a positive control.

**Table 4:** Zone of bacterial growth inhibition (mm) of the EtOAc extract and isolated compounds from the root barks of *G. involucrata* at 1.5 mg/mL.

Samples	Zone of bacterial growth inhibition in mm			
	Gram-positive bacteria	Gram-negative bacteria		
	*S. aureus*	*P. mirabilis*	*E. coli*	*Klebsiella pneumonia*
EtOAc extract	23 ± 0.02	12 ± 0.01	14 ± 0.04	12 ± 0.03
Compound **1**	6 ± 0.01	6 ± 0.01	6 ± 0.02	6 ± 0.04
Compound **2**	8 ± 0.02	6 ± 0.02	11 ± 0.02	6 ± 0.02
Compound **3**	6 ± 0.01	7 ± 0.02	12 ± 0.03	8 ± 0.01
Ciprofloxacin	22 ± 0.03	24 ± 0.03	21 ± 0.02	19 ± 0.02

## Data Availability

The NMR spectra used for the interpretation of compounds in this study are included as supplementary information files. Other data used to support the findings of this study are available from the corresponding author upon request.

## Supplementary Materials

Supplementary Materials Supporting File 1: 1H-NMR spectrum of compound 1. Supporting File 2: 13C-NMR spectrum of compound 1. Supporting File 3: DEPT-135 spectrum of compound 1. Supporting File 4: 1H-NMR spectrum of compound 2. Supporting File 5: 13C-NMR spectrum of compound 2. Supporting File 6: DEPT-135 spectrum of compound 2.

## References

[1] Al-Bari M. A. A., Sayeed M. A., Rahman M. S., Mossadik M. A. (2006). Characterization and antimicrobial activities of phenolic acid derivative produced by *Streptomyces bangladeshiensis*, a novel species collected in Bangladesh. *Research Journal of Medicine and Medical Sciences*.

[2] Khan U. A., Rahman H., Niaz Z. (2013). Antibacterial activity of some medicinal plants against selected human pathogenic bacteria. *European Journal of Microbiology and Immunology*.

[3] Nascimento G. G. F., Juliana L., Paulo C. F., Silva G. L. (2000). Antibacterial activity of plant extracts and phytochemicals on antibiotic resistant bacteria. *Brazilian Journal of Microbiology*.

[4] Hofer E., Quintaes B. R., Reis E. M. F. d. (1999). Emergência da múltipla resistência a antimicrobianos em *Vibrio cholerae* isolados de pacientes com gastroenterite no Ceará, Brasil. *Revista da Sociedade Brasileira de Medicina Tropical*.

[5] Bhandurge P., Rajarajeshwari N., Ganapaty S., Pattanshetti S. (2013). The *Gnidia* genus: a review. *Asian Journal of Biomedical and Pharmaceutical Sciences*.

[6] Peterson B. (1978). *Flora of Tropical East Africa. Thymelaeaceae*.

[7] Sohni Y. R., Mutangadura-Mhlanga T., Kale P. G. (1994). Bacterial mutagenicity of eight medicinal herbs from Zimbabwe. *Mutation Research/Genetic Toxicology*.

[8] Borris R. P., Blaskó G., Cordell G. A. (1988). Ethnopharmacologic and phytochemical studies of the thymelaeaceae. *Journal of Ethnopharmacology*.

[9] Munkombwe N. M., Galebotswe P., Modibesane K., Morebodi N. (2003). Phenylpropanoid glycosides of *Gnidia polycephala*. *Phytochemistry*.

[10] Van-Wyk C. M., Gericke N. (2000). *People’s Plants*.

[11] Getaneh G. (2011). Ethnobotanical study of traditional use of medicinal plants and their conservation status in Ethiopia.

[12] Getachew A., Befikadu U., Amha W. (2017). Systematic review on traditional medicinal plants used for the treatment of malaria in Ethiopia: trends and perspectives. *Malaria Journal*.

[13] Berhanu A., Asfaw Z., Kelbessa E. (2006). Ethnobotany of plants used as insecticides, repellents and antimalarial agents in Jabitehnan district, West Gojjam. *SINET: Ethiopian Journal of Science*.

[14] Singh S. K., Sharma V. K., Kumar Y., Kumar S. S., Sinha S. K. (2009). Phytochemical and pharmacological investigations on mangiferin. *Herbapolonica*.

[15] Kumar S., Kumar V., Rana M., Kumar D. (2012). Enzymes inhibitors from plants: an alternate approach to treat diabetes. *Pharmacognosy Communications*.

[16] Küpeli E., Aslan M., Gürbüz İ., Yesilada E. (2004). Evaluation of *in vivo* biological activity profile of isoorientin. *Zeitschrift für Naturforschung C*.

[17] Ferrari J., Terreaux C., Sahpaz S., Msonthi J. D., Wolfender J.-L., Hostettmann K. (2000). Benzophenone glycosides from Gnidia involucrata. *Phytochemistry*.

[18] Ferrari J., Terreaux C., Kurtán T. (2003). Isolation and on-line LC/CD analysis of 3, 8″-linked biflavonoids from *Gnidia involucrata*. *Helvetica Chimica Acta*.

[19] Brand-Williams W., Cuvelier M. E., Berset C. (1995). Use of a free radical method to evaluate antioxidant activity. *LWT—Food Science and Technology*.

[20] Wang H., Gao X. D., Zhou G. C., Cai L., Yao W. B. (2008). In vitro and in vivo antioxidant activity of aqueous extract from *Choerospondias axillaris* fruit. *Food Chemistry*.

[21] Nagatsu A. (2004). Investigation of anti-oxidative compounds from oil plant seed. *FABAD Journal of Pharmaceutical Sciences*.

[22] Gülçin İ., Huyut Z., Elmastaş M., Aboul-Enein H. Y. (2010). Radical scavenging and antioxidant activity of tannic acid. *Arabian Journal of Chemistry*.

[23] Wayne P. A. (1997). *Performance Standards for Antimicrobial Disc Susceptibility Test, Approved Standard: M2-A6*.

[24] Obdak A., Zielinska D., Rzepkowska A., KoBohyn-Krajewska D. (2017). Comparison of antibacterial activity of *Lactobacillus plantarum* strains isolated from two different kinds of regional cheeses from Poland: Oscypek and Korycinski cheese. *BioMed Research International*.

[25] Deganhardt J., Gershenzon J., Baldwin I. T., Kessler A. (2003). Attracting friends to feast on foes: engineering terpene emission to make crop plant more attractive to herbivore enemies. *Current Opinion in Biotechnology*.

[26] Jieru G., Jinwen Z., Penghua S. (2012). Two new diterpenoids from the buds of *Wikstroemiachamaedaphne*. *Molecules*.

[27] Michal K., Vitthal W., I-Wen L. (2016). Antiallergic phorbol ester from the seeds of *Aquilaria malaccensis*. *International Journal of Molecular Sciences*.

[28] Biancotto A., Grivel J. C., Gondois-Rey F. (2004). Dual role of prostratin in inhibition of infection and reactivation of human immunodeficiency virus from latency in primary blood lymphocytes and lymphoid tissue. *Journal of Virology*.

[29] Korin Y. D., Brooks D. G., Brown S., Korotzer A., Zack J. A. (2002). Effects of prostratin on T-cell activation and human immunodeficiency virus latency. *Journal of Virology*.

[30] Gulakowski R., McMahon J., Buckheit R., Gustafson K., Boyd M. (1997). Antireplicative and anticytopathic activities of prostratin, a non-tumor-promoting phorbol ester, against human immunodeficiency virus (HIV)1. *Antiviral Research*.

[31] Szallasi Z., Blumberg P. M. (1991). Prostratin, a nonpromoting phorbol ester, inhibits induction by phorbol 12-myristate 13-acetate of ornithine decarboxylase, edema, and hyperplasia in CD-1 mouse skin. *Cancer Research*.

[32] Jain R., Mittal M. (2012). Naringenin, a flavanone from the stem of *Nyctanthes arbortristis*. *International Journal of Biology, Pharmacy, and Applied Sciences*.

[33] Olszowy M., Dawidowicz A. L. (2018). Is it possible to use the DPPH and ABTS methods for reliable estimation of antioxidant power of colored compounds?. *Chemical Papers*.

