# Supplementary material for: Chemical Constituents of Root Barks of Gnidia involucrata and Evaluation for Antibacterial and Antioxidant Activities
Source: J Trop Med. 2019 Aug 14;2019:8486214. doi: 10.1155/2019/8486214 (PMC6710788; doi:10.1155/2019/8486214)
Supplement: Supplementary Materials — Supporting File 1: 1H-NMR spectrum of compound 1. Supporting File 2: 13C-NMR spectrum of compound 1. Supporting File 3: DEPT-135 spectrum of compound 1. Supporting File 4: 1H-NMR spectrum of compound 2. Supporting File 5: 13C-NMR spectrum of compound 2. Supporting File 6: DEPT-135 spectrum of compound 2. [file 8486214.f1.docx]

Supporting Information files

Supporting file 1: ^1^H-NMR spectrum of compound 1

Supporting file 2: ^13^C-NMR spectrum of compound 1

Supporting file 3: DEPT-135 spectrum of compound 1

Supporting file 4: ^1^H-NMR spectrum of compound 2

Supporting file 5: ^13^C-NMR spectrum of compound 2

Supporting file 6: DEPT-135 spectrum of compound 2

Supporting file 7**:** ^13^C-NMR spectrum of compound 3
